# Anesthetic management of hip fracture in geriatric patient with respiratory and heart failure using pericapsular nerve group block: A case report

**DOI:** 10.1097/MD.0000000000029478

**Published:** 2022-06-03

**Authors:** Zejun Niu, Xiaolin Xu, Haichen Chu, Jihui Yin

**Affiliations:** Department of Anesthesiology, The Affiliated Hospital of Qingdao University, Qingdao, China.

**Keywords:** heart failure, hip fracture, pericapsular nerve group block, postoperative complications, respiratory failure

## Abstract

**Introduction:**

Hip fracture with severe cardiopulmonary and cerebral dysfunction is a relatively common problem in the elderly population and poses a great challenge to anesthetic management. Pericapsular nerve group (PENG) block combined with nerve blocks of the hip region has recently attracted significant interest from anesthesiologists, and very few reports on its anesthetic management exist.

**Patient concerns:**

Patient suffered from the right femoral neck fracture, combined with respiratory failure, heart failure, moderate-to-severe pulmonary hypertension, cerebral infarction, atrial fibrillation, and cognitive dysfunction.

**Diagnosis:**

Because of right femoral neck fracture, artificial femoral head replacement was scheduled for this patient.

**Interventions:**

Ultrasound-guided PENG block combined with sacral plexus, thoracic 11 to 12 paravertebral block, and lateral femoral cutaneous block were performed to a high-risk elderly patient.

**Outcomes:**

The patient successfully received artificial femoral head replacement with our effective anesthesia techniques and no postoperative complication was reported.

**Conclusions:**

Among elderly patients with multiple organ dysfunction undergoing hip surgery, PENG block combined with nerve blocks of the hip region is an ideal anesthesia method. This case demonstrated that these regional analgesia techniques had a stable hemodynamic process, satisfactory anesthetic effect, effective postoperative analgesia, and no effect on postoperative cognitive function. Further studies are needed to determine the appropriate doses of local anesthetics in the elderly with multiple organ system failure to reduce delayed local anesthesia systemic toxicity.

## 1 Introduction

Hip fractures are associated with increased morbidity and mortality, especially in elderly patients.^[1,2]^ Perioperative anesthesia management is a challenge in the geriatric surgical population, with a decline in pulmonary or cardiac functional status. Effective regional analgesia for pain originating from the hip after a fracture or during surgery can be described as elusive.^[3]^ Although some nerve blocks have been used in hip fracture surgeries, such as fascia iliac block,^[4,5]^ femoral nerve block,^[6]^ and lumbar plexus block,^[7]^ these regional blocks do not fully cover the obturator nerve and accessory obturator nerve (AON).^[8]^ One of the difficulties of effective regional analgesia and anesthesia for hip surgery is the complex innervation of the joint as it comes from multiple nerves. Pericapsular nerve group block (PENG) is a regional anesthetic technique described in 2018,^[9]^ developed primarily in total hip arthroplasty for postoperative analgesia with motor-sparing benefits. Therefore, PENG block in combination with other regional analgesia techniques for elderly patients with multiple organ dysfunction, which can block the innervation of the hip region may be an appropriate anesthetic method.

## 2 Case report

A 92-year-old female (American Society of Anesthesiologists status III) was scheduled to undergo artificial femoral head replacement for a right femoral neck fracture. Preoperative complications included atrial fibrillation, coronary heart disease, heart failure, pneumonia, respiratory failure, cerebral infarction, pulmonary hypertension (moderate-to-severe), valvular disease (severe tricuspid regurgitation), and hypoproteinemia. The brain natriuretic peptide test result was 5958.00 pg/ml. Blood gas analysis showed an oxygen partial pressure of 52 mm Hg, a blood glucose of 8.5 mmol/L and HGB 76 g/L. Echocardiography revealed severe tricuspid regurgitation, pulmonary artery hypertension (PASP: 65 mm Hg), left atrial enlargement, right atrial enlargement, and right ventricular enlargement. The VAS scores ranged from 9 to 10 when the patient slightly moved her injured limb slightly.

The patient underwent standard monitoring and received mask oxygen (5 L/min). Radial artery catheterization was performed under local anesthesia and sufentanil (3 μg) was administered intravenously for mild analgesia. Dexmedetomidine was continuously infused for 20 minutes at a rate of 0.6 μg/kg, then it was infused at the rate of 0.1 to 0.2 mg/kg/h. The bispectral index remained between 40 and 60 when propofol was continuously administered intravenously.

**Figure 1:**
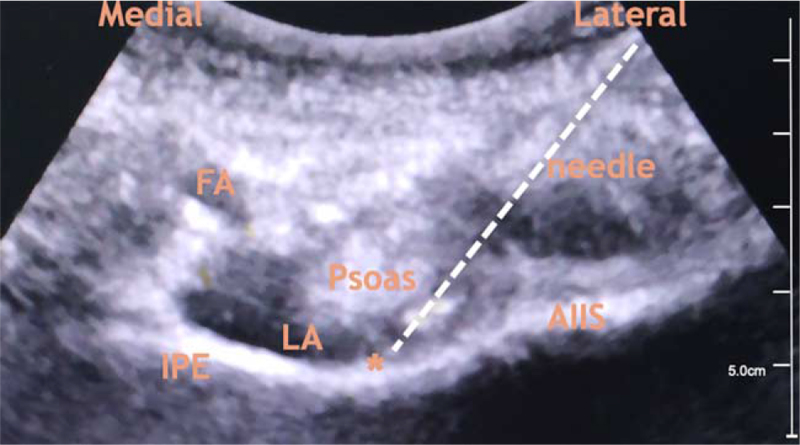
Ultrasonographic view during PENG block. AIIS = anterior inferior iliac spine, FA = femoral artery, IPE = iliopubic eminence, LA = local anesthetic, PENG = pericapsular nerve group block. Asterisk (_∗_): target for local anesthetic injection.

Under sterile conditions, a low-frequency curvilinear probe of ultrasound probe (Logiq E Ultrasound Machine, GE, USA) was initially placed in a transverse plane over the anterior inferior iliac spine and was then rotated parallel to the pubic ramus to obtain a short-axis view of the iliopsoas muscle and tendon lying on the ramus pubic adjacent to the iliopubic eminence. A 22 G, 80 mm insulated block needle (Stimuplex; B Braun, Germany) was inserted in-plane in a lateral-to-medial direction to place the tip in the musculofascial plane between the psoas tendon and the pubic ramus (Fig. 1). A total of 20 ml 0.3% ropivacaine (AstraZeneca, Sweden, Wuxi) was injected slowly in 5 ml increments with intermittent aspiration and under constant ultrasound surveillance for adequate fluid spread. Ultrasound-guided sacral plexus block (0.3% ropivacaine, 15 ml), thoracic 11 to 12 paravertebral block (0.3% ropivacaine, 10 ml), and lateral femoral cutaneous block (0.3% ropivacaine, 5 ml) were performed.

Thirty minutes later after the regional blocks, we confirmed that the blocks worked well using the pinprick test. The patient was then placed in the left decubitus position, and the VAS scores was <2 during this process. The intraoperative hemodynamics were stable and the total dosage of sufentanil was 10 μg.

No additional analgesia was administered in the post-anesthesia care unit. The quality of recovery score (QoR-15) at postoperative 24-hour was 125, and the VAS score at postoperative 48 hours was less than 3 points. The lower limb muscle strength was 3/5 on the postoperative day 1. The patient was discharged from the hospital on the postoperative day 5. No related postoperative complications, such as delirium, were observed. The patient satisfaction score was 10/10.

The study was approved by the Ethics Committee of the affiliated hospital of Qingdao University. Written informed consent was obtained from the patient for publication of the case details.

## 3 Discussion

Hip fracture is called the last fracture in life because its complications and mortality rates of this fracture are very high. Choosing an appropriate method of anesthesia is of great significance for the perioperative rehabilitation of elderly patients with hip fractures. Commonly used anesthesia methods for patients with hip fractures include general anesthesia combined with spinal anesthesia and general anesthesia combined with nerve block, such as femoral nerve block and fascia iliac block and so on. In elderly patients with severe cardiopulmonary insufficiency, appropriate anesthesia plays an important role in the recovery of postoperative patients and reduces complications.

The PENG block was first reported at the University of Toronto scholar Girón-Arango in 2018.^[9]^ The advantage of this block is that it can block the hip joint branches of the femoral, obturator, and AONs, while having a lesser effect on the strength of the quadriceps muscle. Although this patient had many complications (severe pulmonary hypertension, type I respiratory failure, pulmonary infection, cerebral infarction, atrial fibrillation, severe tricuspid regurgitation, and hypoproteinemia), early surgery is important, with decreased complication and mortality rates in patients receiving surgery within 48 hours.^[10,11]^ If intubated general anesthesia is used alone, there are several aspects that anesthesiologists have to deal with, such as severe fluctuations in intraoperative hemodynamics, length of stay in the ICU, and changes in postoperative cognitive function. We chose nerve blocks combined with general anesthesia because nerve blocks provided optimal perioperative analgesia and that general anesthesia also provided sedation and appropriate analgesia. We did not perform endotracheal intubation because the patient had comorbidities such as lung infection, which there is also a risk of difficulty in extubation after surgery. If a spinal anesthesia is used, there is a possibility that the spinal puncture may fail due to difficulty in positioning. Therefore, the ideal method of anesthesia is to preserve spontaneous breathing, block the relevant nerves during hip surgery, and reduce circulation fluctuations. Simultaneously, the consumption of sufentanil, propofol and related complications will be decreased.

In this case, we performed PENG block (0.3% ropivacaine 20 ml), lateral femoral cutaneous nerve block (0.3% ropivacaine 5 ml), sacral plexus block (0.3% ropivacaine 15 ml) and thoracic 11 to 12 paravertebral nerve block (0.3% ropivacaine 10 ml), combined with BIS index-guided propofol sedation. The total dose of sufentanil was 10 μg during the operation, which also reflects the satisfactory effect of the nerve blocks. Hemodynamics were stable during the operation, and the effect of anesthesia was satisfactory. The nerves that innervate the artificial femoral head replacement may include branches of the femoral nerve, obturator nerve, AON, lateral femoral cutaneous nerve, sciatic nerve, and thoracic 11 to 12 spinal nerve.^[12]^ Although we blocked innervation of the hip region as much as possible and provided suitable anesthesia and analgesia for the surgery, it is necessary to strengthen the perioperative monitoring in elderly patients with multiple organ system failure. Injection of high-volume anesthetics should always raise concerns about delayed local anesthetic systemic toxicity.

Although PENG block, sacral plexus block, and paravertebral nerve blocks provide optimal analgesia, the risk of local anesthetic system toxicity and nerve damage should also be considered. The PENG block has several benefits. First, the PENG block can significantly relieve the hip pain by placing the patient in the lateral decubitus position and making the following blocks easier to perform. Second, the PENG block did not affect quadriceps muscle strength after surgery^[13]^ or postoperative cognitive function. This choice of anesthesia may be an attractive anesthesia method because it can promote early rehabilitation of patients and avoid the hemodynamic changes caused by general anesthetics and intubation.

In summary, for elderly hip fracture patients with preoperative respiratory failure, heart failure and other organ complications, the PENG block combined with other regional techniques (lateral femoral cutaneous nerve block, sacral plexus block, and thoracic 11–12 paravertebral nerve block) are advantageous because of the reduced need for systemic medications that may alter the patient's sensorium or cause cardiopulmonary depression. This anesthetic method can provide effective analgesia, stable hemodynamics, and little impact on cognitive function. Large-scale randomized controlled prospective studies in elderly patients with severe comorbidities undergoing hip surgery are warranted to validate the efficacy and superiority of the PENG block combined with different nerve blocks over other anesthetic techniques.

## Acknowledgments

We especially thank Lu Liu and Wenyan Zhou for being helpful in conducting and finish this research.

## Author contributions

**Conceptualization:** Zejun Niu and Haichen Chu.

**Data curation:** Xiaolin Xu, ZeJun Niu.

**Formal analysis:** Haichen Chu, Zejun Niu and Jihui Yin.

**Funding acquisition:** ZeJun Niu.

**Investigation:** Xiaolin Xu, ZeJun Niu.

**Methodology:** Haichen Chu, ZeJun Niu.

**Project administration:** Jihui Yin.

**Supervision:** Haichen Chu.

**Validation:** Jihui Yin.

**Writing – original draft:** Jihui Yin, ZeJun Niu.

**Writing – review & editing:** ZeJun Niu.

**Writing:** Zejun Niu and Haichen Chu.

## References

[1] Chen AF Barrington JW Duwelius PJ. Trends of femoral neck fracture treatment using total hip arthroplasty: reported from the American Joint Replacement Registry. J Am Acad Orthop Surg 2022;30:e44–50. 34192715 10.5435/JAAOS-D-21-00132

[2] Smolle MA Horlesberger N Maurer-Ertl W Puchwein P Seibert FJ Leithner A. Periprosthetic fractures of hip and knee-A morbidity and mortality analysis. Injury 2021;52:3483–8. 33536128 10.1016/j.injury.2021.01.015

[3] Garip L Balocco AL Van Boxstael S. From emergency department to operating room: interventional analgesia techniques for hip fractures. Curr Opin Anaesthesiol 2021;34:641–7. 34325461 10.1097/ACO.0000000000001046

[4] Kolodychuk N Krebs JC Stenberg R Talmage L Meehan A DiNicola N. Fascia Iliaca Blocks performed in the emergency department decrease opioid consumption and length of stay in patients with hip fracture. J Orthop Trauma 2022;36:142–6. 34294666 10.1097/BOT.0000000000002220

[5] Schulte SS Fernandez I Van Tienderen R Reich MS Adler A Nguyen MP. Impact of the Fascia Iliaca Block on pain, opioid consumption, and ambulation for patients with hip fractures: a prospective, randomized study. J Orthop Trauma 2020;34:533–8. 32358477 10.1097/BOT.0000000000001795

[6] Uysal AI Altiparmak B Yasar E. The effects of early femoral nerve block intervention on preoperative pain management and incidence of postoperative delirium geriatric patients undergoing trochanteric femur fracture surgery: a randomized controlled trial. Ulus Travma Acil Cerrahi Derg 2020;26:109–14. 31942744 10.14744/tjtes.2019.78002

[7] Schonwald G Skipper GE Smith DE Earley PH. Anesthesiologists and substance use disorders. Anesth Analg 2014;119:1007–10. 25329014 10.1213/ANE.0000000000000445

[8] Deegan CA Murray D Doran P Ecimovic P Moriarty DC Buggy DJ. Effect of anaesthetic technique on oestrogen receptor-negative breast cancer cell function in vitro. Br J Anaesth 2009;103:685–90. 19776028 10.1093/bja/aep261

[9] Giron-Arango L Peng PWH Chin KJ Brull R Perlas A. Pericapsular Nerve Group (PENG) block for hip fracture. Reg Anesth Pain Med 2018;43:859–63. 30063657 10.1097/AAP.0000000000000847

[10] Boddaert J Raux M Khiami F Riou B. Perioperative management of elderly patients with hip fracture. Anesthesiology 2014;121:1336–41. 25299743 10.1097/ALN.0000000000000478

[11] Sheehan SE Shyu JY Weaver MJ Sodickson AD Khurana B. Proximal femoral fractures: what the orthopedic surgeon wants to know. Radiographics 2015;35:1563–84. 26186669 10.1148/rg.2015140301

[12] Sakamoto J Manabe Y Oyamada J. Anatomical study of the articular branches innervated the hip and knee joint with reference to mechanism of referral pain in hip joint disease patients. Clin Anat 2018;31:705–9. 29577432 10.1002/ca.23077

[13] Allard C Pardo E de la Jonquiere C. Comparison between femoral block and PENG block in femoral neck fractures: a cohort study. PLoS One 2021;16:e0252716. 34086782 10.1371/journal.pone.0252716 PMC8177466

